# T cell decision-making decodes the dynamic antigenic landscape

**DOI:** 10.3389/fimmu.2026.1806850

**Published:** 2026-06-01

**Authors:** Inbal Eizenberg-Magar, Lior Dayan, Benny Chain, Yaron E. Antebi

**Affiliations:** 1 Department of Molecular Genetics, Weizmann Institute of Science, Rehovot, Israel; 2 Division of Infection and Immunity, UCL, London, United Kingdom

**Keywords:** antigen dynamics, immune decision, kinetic proofreading, signal decoding, systems immunology, T cell activation, T cell receptor signaling, temporal signal processing

## Abstract

The adaptive immune system continuously encounters antigens from a wide range of sources, including pathogens, self-tissues, tumors, and environmental agents. While extensive studies have characterized how lymphocytes respond to antigen binding, most experimental frameworks consider the antigenic environment as static. In reality, antigen levels can fluctuate dramatically across a wide range of temporal and spatial scales. In this review, we examine how the dynamics of antigen presentation, ranging from molecular binding events to organism-level exposure, affect T cell activation and fate. We discuss the cellular and molecular mechanisms that allow T cells to detect and respond to changes in antigen concentration over timescales from seconds to days. These include kinetic proofreading of TCR signaling, frequency-dependent decoding in intracellular signaling networks, and population-level feedback circuits involving effector and regulatory T cells. Theoretical and experimental evidence suggests that T cells are tuned not only to antigen quantity but also to its rate of change, with implications for tolerance, immune activation, and memory formation. We highlight how manipulating the dynamics of antigen exposure, such as through controlled vaccine delivery, can modulate immune responses and suggest that incorporating temporal features into immunological models may improve our understanding of immune decision-making and inform therapeutic strategies.

## Introduction

The immune system is exposed to an enormous variety of antigens derived from microbes, self, altered self (e.g., cancers), and innocuous environmental components such as food. Adaptive immunity in vertebrates decides qualitatively and quantitatively when and how to respond to these stimuli using a hyper-diverse set of molecules on the surface of lymphocytes, the B and T cell antigen receptors (BCR and TCR). These receptors can bind antigens with high sensitivity and specificity, discriminating subtle molecular differences between antigens, as well as differences in concentration. The binding of an antigen can initiate a complex signaling pathway in the responding lymphocytes, triggering widespread changes in gene transcription, translation, and degradation, and ultimately inducing diverse functional outputs, including entry into the cell cycle, cellular differentiation, and death. Immunologists have studied the dynamic series of events that follow antigen binding in enormous molecular detail. However, the majority of studies, both *in vitro* and *in vivo*, have focused on the dynamics of the host response but have taken a much more static perspective on the antigenic environment. Experimentally, antigens are often delivered as a single bolus, and little attention is paid to the rate of antigen removal or clearance (antigen pharmacokinetics). This approach allows for investigating the impact of antigen dose, but the temporal pattern of antigen exposure is fixed by design. Thus, while the conceptual importance of antigen dynamics in self/non-self discrimination has been recognized (1, 2), their broader impact on immune responses is often overlooked. An additional layer of complexity arises from the intrinsic heterogeneity of T cell populations and the diverse physiological contexts in which they operate. Differences in TCR affinity, co-receptor expression, signaling protein abundance, and differentiation state can significantly influence how individual T cells interpret antigen dynamics (3–5). For instance, naïve, effector, and memory T cells often exhibit distinct activation thresholds and signaling kinetics, and their responses can vary across TCR-ligand pairs even when antigen concentration is similar (6–8). Furthermore, the physiological context, including the presence of co-stimulatory molecules and pro-inflammatory cytokines, tunes these thresholds and integrates with antigen signaling to determine the magnitude of T cell responses (9–11). Therefore, antigen dynamics are interpreted within a broader regulatory landscape defined by cell-intrinsic properties and microenvironmental cues to shape the overall population response. In this review, we consider first the biological context in which antigen concentration changes, which span vastly different time scales. In the second part of the review, focusing primarily on T cells, we consider possible mechanisms by which the immune system can measure and respond to antigen temporal gradients. Finally, we discuss the potential implications of these features for preventative or therapeutic manipulation of immunity.

## The antigenic environment is dynamic at different temporal and organizational scales

**Figure 1:**
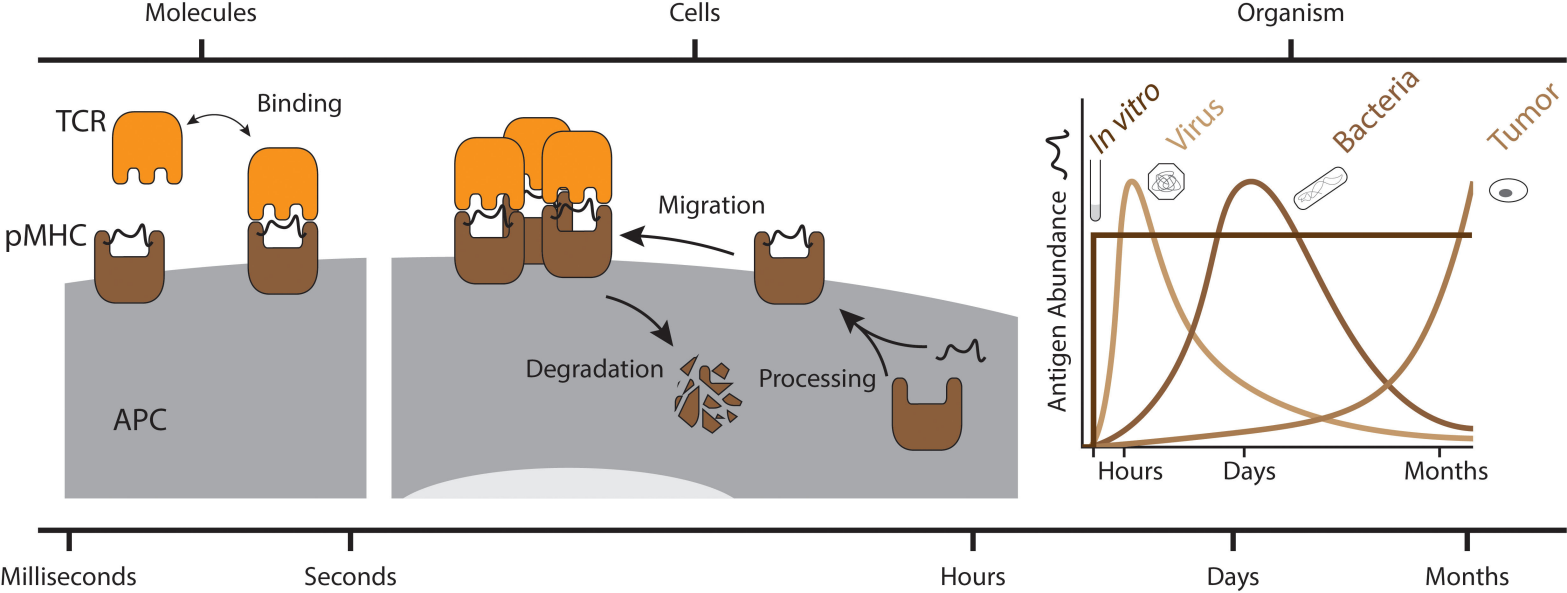
The antigenic landscape is intrinsically dynamic across different timescales. Antigen exposure (right), antigen processing and presentation (middle), and reversible TCR-pMHC binding (left) shape the temporal antigen landscape across different organizational and temporal scales.

We consider the dynamics of the antigenic environment at three levels of organizational complexity, associated with different temporal dynamic scales (illustrated in **Figure 1**). A fourth level from which to consider the dynamics of antigen exposure could be at the population (epidemiological level), however, as co-evolution of pathogens and host immunity has received a lot of attention it is not considered further in this review.

**Organismal antigen dynamics** (**Figure 1**, right). At the level of the whole organism, antigen levels are determined by the balance between production by the antigen source (e.g., infecting microorganism) and removal by the host. In the context of exposure to infectious microorganisms, the replication rate and, therefore, the increase in antigen concentration is often exponential and can occur over vastly different time ranges. Some bacteria and viruses, including E. coli and other common pathogens, have doubling times in the order of 20 minutes to a few hours. For example, the number of SARS-COV-2 virions can increase by 10^5^ in a single day (an effective doubling time of 1–2 hours) (12). However, the important human pathogen Mycobacterium tuberculosis divides only every 30 days or longer. The examples above are all examples of more-or-less ‘acute’ antigen exposure. Low-level exposure to chronic viruses, symbiotic components of the microbiome, food antigens, or environmental allergens may occur over an extended period of months or even years. Indeed, the recognition that low-dose chronic exposure could give a different outcome than similar total levels of antigen delivered in one pulse gave rise to the concept of low zone tolerance (13), one of the earliest recognitions that antigen dynamics is a key determinant of response. Similarly, cancer cells carrying potential neoantigens often grow slowly over months or years. The rate of growth varies considerably and can depend on cancer type (14, 15). The slow growth of tumor cells has been proposed to be one of the factors facilitating immune evasion (16). However, slow rates of exposure are not always tolerogenic. Allergen exposure is typically low dose, but builds up over periods of weeks or months. The factors that determine sensitization versus tolerization remain much debated. At the other extreme, vaccines are usually delivered as an instantaneous pulse of antigen at high concentrations. While free protein is very rapidly removed from circulation, it has been known for over a century that one function of adjuvants is to act as antigen depots, decreasing the rate of antigen disappearance and dramatically enhancing the immune response.

**Tissue and cellular antigen dynamics** (**Figure 1**, middle). At the tissue level, the microenvironment acts as a spatial filter that shapes antigen availability. Within lymphoid organs and peripheral tissues, antigen presentation is shaped by the spatial organization and turnover of antigen-presenting cells (17, 18), the migration and scanning behavior of T cells, and the architecture of the tissue microenvironment (19–22). As T cells migrate through tissues, they experience antigen not as a continuous signal but rather as a series of discrete, stochastic encounters with antigen-presenting cells. The frequency and duration of these interactions, as well as local antigen distribution, determine how T cells integrate signals. The effective antigen dose perceived by the cell, therefore, reflects tissue-scale migration dynamics (23–25).

Within a tissue, at the cellular level, antigens require prior uptake, processing, and presentation as pMHC complexes by antigen-presenting cells in order to be recognized by T cells. The biology of these cells, therefore, determines the actual kinetics of how much antigen is ‘seen’ by the T cell, dynamically shaping the antigenic signal, even at short timescales of minutes to hours over which the overall antigen levels may remain approximately constant. In most cases, the kinetics of the internal biochemical processes (the rates at which antigen-loaded MHC complexes are formed, exported to the cell surface, and then internalized and destroyed) are not known with precision but may vary depending on the physiological status of the cell (26–31). The turnover kinetics of the antigen-presenting cells themselves, which include a diverse population of professional presenting cells, and many other cell types that can act as facultative presenting cells via Class I or Class II MHC, impose another level of dynamism on the antigen landscape. Processing and presentation by different cells (32) and of different antigens (33–35) can lead to different kinetics of pMHC formation. In addition, the stability of pMHC complexes can vary widely depending on epitope sequence and structure, influencing the duration of antigen presentation on the cell surface and thereby modulating T cell recognition (36). Similarly, the rate at which antigen disappears from the surface of cells, whether by internalization, TCR-mediated uptake by trans-endocytosis, sometimes described as ‘grazing’ (37–42), or cell turnover, may also regulate T cell activation (43, 44). At the level of antigen presentation, several factors determine the rate at which pMHC is displayed to T cells at the immunological synapse. Free pMHC and TCR move rapidly by lateral diffusion in the plane of the membrane (45), but the distribution and rate can be affected by its lipid composition (46–49). The dynamic interaction between pMHC and TCR is also determined by the specialized composition of the immunological synapse (50).

In addition to binding kinetics, the physical properties of the immunological synapse can profoundly influence T cell activation. Factors such as lateral mobility of membrane proteins (51), nanoscale spacing of pMHC complexes (52), substrate rigidity (53), and the topographical constraints of the glycocalyx (54) all modulate TCR triggering and signaling. These physical parameters affect receptor accessibility and the efficiency of mechanical force transmission across the TCR-pMHC bond (55, 56). While this review focuses primarily on the temporal dynamics of antigen dose and duration, these biophysical features represent an important, complementary layer in the interpretation of dynamic inputs.

**Molecular antigen dynamics** (**Figure 1**, left). Even at shorter timescales, where the overall level of pMHC on an antigen-presenting cell remains approximately constant, a distinct layer of dynamics arises from the interaction of individual pMHC and TCR molecules. This engagement is an extremely rapid process, with individual pMHC complexes binding and unbinding in the timescale of seconds (57, 58). Recent single-molecule studies have further refined this view, demonstrating that even a single agonist pMHC complex can be sufficient to trigger a functional T cell response in certain contexts (45, 59, 60). However, the precise mechanisms governing these molecular dynamics remain an area of active debate. In addition to binding kinetics, models of T cell activation invoke contributions from serial engagement or rebinding, the spatial distribution of ligands within the immunological synapse, and mechanical forces that can modulate TCR-pMHC bond lifetimes (61–63). Within this complex physical landscape, the dwell-time of the TCR-pMHC interaction remains a key determinant of the immunological outcome. For example, the potency of different peptides has been correlated with the off-rate of peptide binding rather than overall equilibrium affinity (64). More generally, each individual binding event only triggers a limited intracellular signaling response. The T cell integrates these repeated small signals, arising either from receptor binding/unbinding/rebinding events or from the recruitment of an increasing number of TCRs to the immunological synapse (57, 65). T cells, therefore, respond to a stream of repeated small stimuli, more reminiscent of a high-frequency oscillation than a static antigen pulse. While a comprehensive analysis of these biophysical mechanisms and their spatial organization is beyond the scope of this review, they represent an important layer of complexity in how T cells interpret dynamic antigenic inputs (62, 66).

In summary, the dynamics of the interaction between antigens and T cells are shaped by a variety of biological processes, including antigen exposure, antigen processing and presentation, and TCR-pMHC binding. These processes generate temporal patterns that span a broad range of timescales, from fractions of a second to months or even years. In the following section, we examine the mechanisms by which the adaptive immune system decodes antigen dynamics in addition to absolute antigen concentrations.

## Multi-scale mechanisms for recognizing rates of stimulation

**Figure 2:**
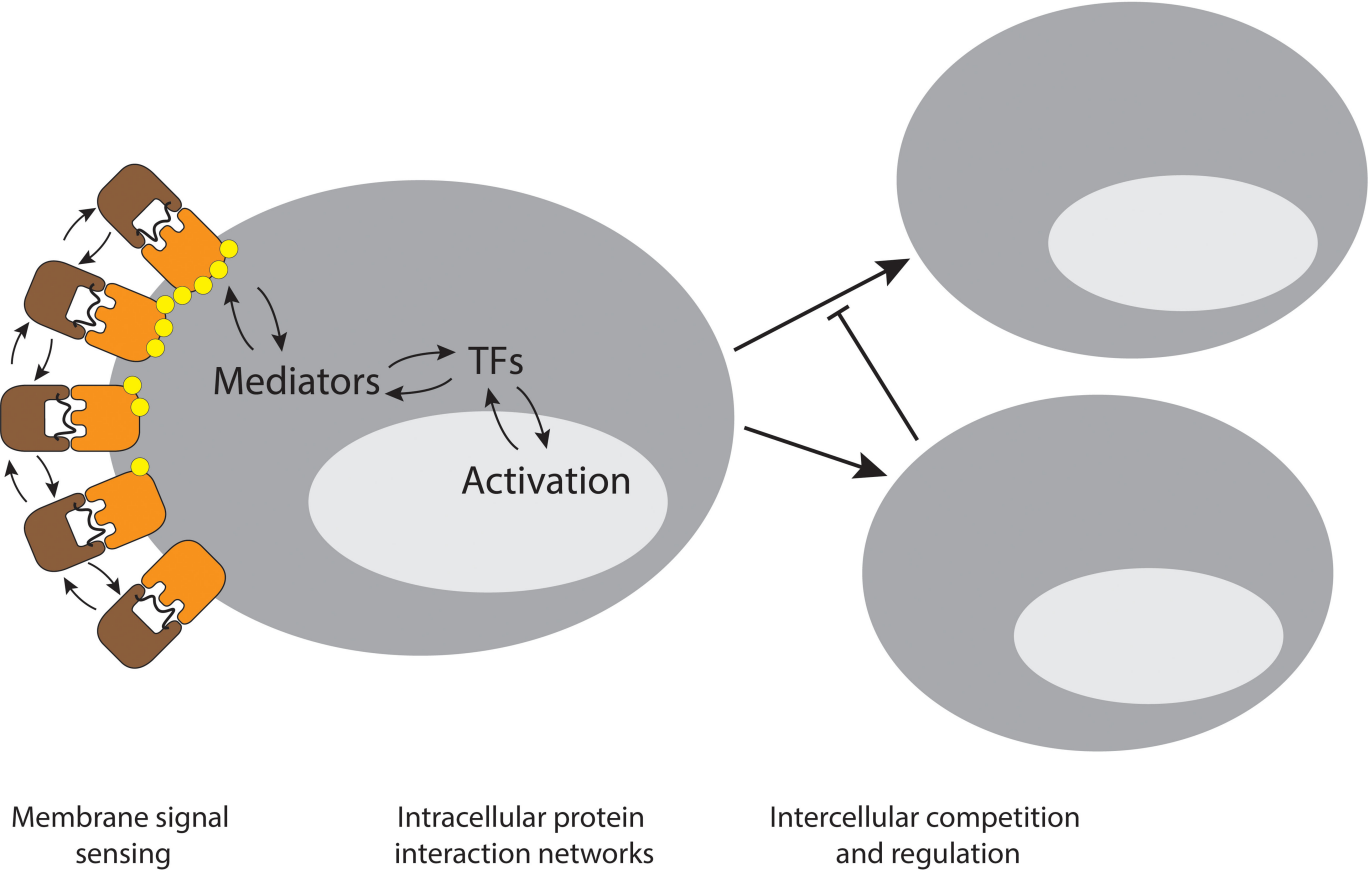
Multiscale mechanisms for decoding antigen dynamics. At the membrane, kinetic proof-reading, which requires multiple reversible phosphorylation events on the TCR complex, filters out short-lived TCR-pMHC interactions, conferring sensitivity to binding duration on the scale of seconds or subseconds (left). Intracellular signaling networks involving mediators, transcription factors (TFs), and other dynamic reversible interactions act as temporal decoders that respond to the frequency and duration of signals over minutes to hours (middle). At the population level, intercellular interactions, such as competition for antigen and activation of regulatory T cell-mediated feedback, modulate responses over hours to days, enabling sensitivity to slow or cumulative changes in antigen availability (right).

We have reviewed above the different dynamics of antigen stimulation and the evidence that these dynamics influence subsequent immune responses. In the following section, we explore possible mechanisms by which the dynamics of antigens, and not just their steady-state concentration, may regulate T cell activation and differentiation. We propose that different biological mechanisms will likely be required to respond to different dynamic ranges. The most well-explored example of sensing the short timescale dynamics of TCR binding is the ‘kinetic-proof-reading’ (KPR) model of T cell signaling (**Figure 2**, left). KPR was introduced in biology to account for the high fidelity of DNA replication (67). It was subsequently applied to T cell signaling to explain how TCRs accurately discriminate between peptide ligands with similar affinities in a concentration-independent manner (68, 69).

In this model, binding of the TCR to pMHC triggers a sequence of phosphorylation events that ultimately lead to activation of the downstream pathway. Phosphorylation is rapidly reversed in the absence of binding, requiring the formation of a stable complex. KPR has a strong non-linear damping effect on the downstream signal, such that short binding events are disproportionately silenced. Support for the KPR model came from experiments using peptide variants with different off-rates, leading to distinct dwell times. However, these differences in off-rates were also accompanied by changes in overall affinity (64). To identify the specific effect of temporal dynamics, a different approach was required to directly control TCR phosphorylation at a fast timescale.

**Figure 3:**
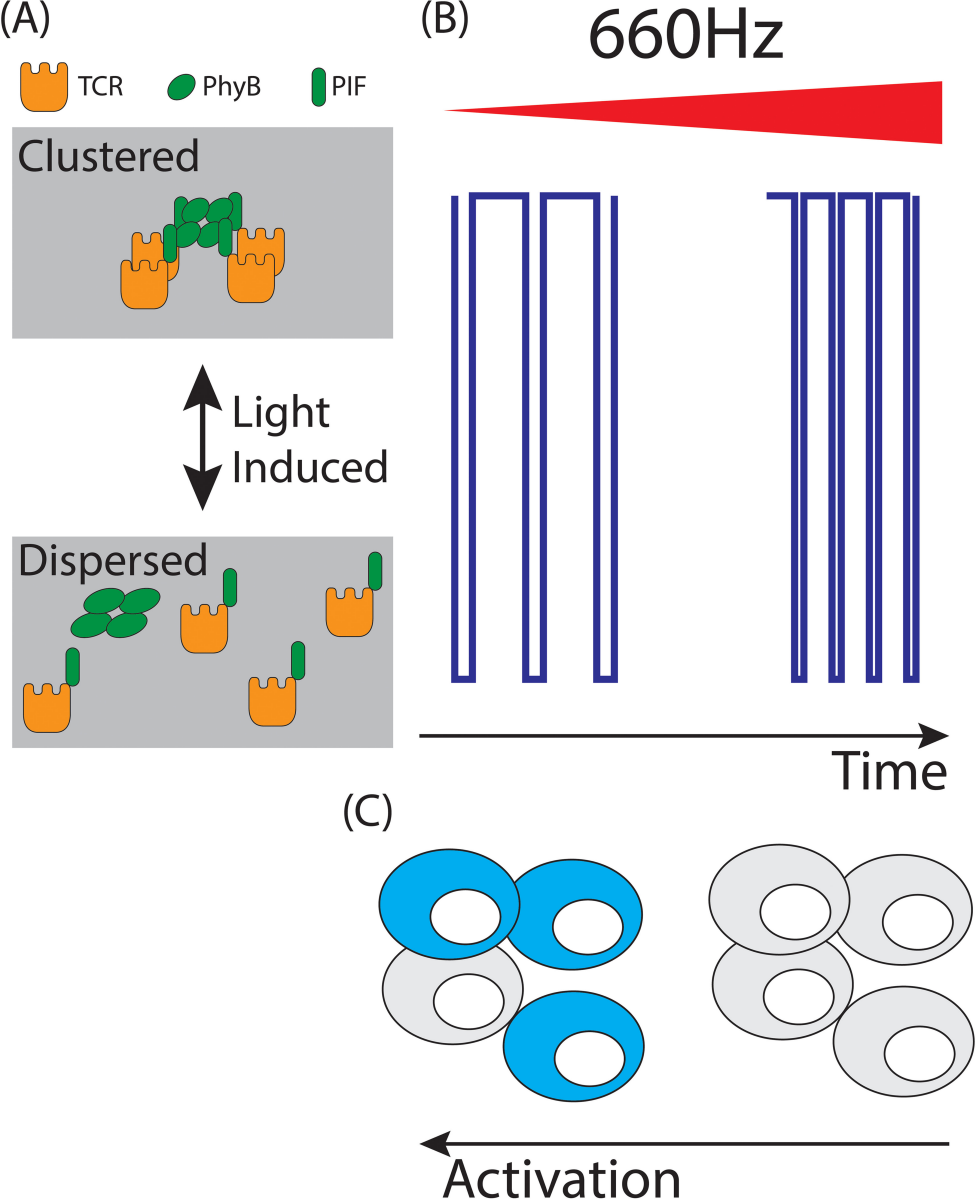
Optogenetic control of TCR clustering allows experimental manipulation of TCR stimulation kinetics in the second or subsecond timescale. PhyB, attached to the TCR, cycles between a PIF binding and non-binding state in a light-dependent manner **(A)**. The rate of cycling, but not the relative proportion of time in the binding and non-binding state is determined by the light intensity **(B)**. At low intensity, the cycling rate is low (i.e. both on and off rates are low) allowing longer ‘on’ dwell times and leading to subsequent T cell activation. At high intensity, the cycling rate is fast, leading to low ‘on’ dwell times, and no cell activation **(C)**. The threshold for activation is around 8 seconds.

Experimental manipulation of TCR-pMHC dynamics at sub-second to minute timescales is challenging. Currently, antigen recognition is thought to occur primarily through interactions of monomeric TCRs with individual pMHC complexes (45, 70). However, as T cell signaling can also be artificially triggered by clustering of TCRs at the membrane, several recent studies have explored optogenetic tools to control receptor proximity with high temporal precision. In these systems, light-sensitive proteins undergo conformational changes in response to specific wavelengths, enabling reversible dimerization of target proteins (71–73). Yousefi et al. (74) exploited the ability of the Phytochrome B protein (PhyB) to continuously cycle between a phytochrome interacting factor (PIF)-binding state and a non-binding state in response to red light (**Figure 3A**). Adjusting the light intensity determines the cycling rate and thus precisely controls the clustering half-life of TCRs (**Figure 3B**) while maintaining a constant ratio between clustered and dispersed receptors. By using PhyB in place of pMHC to induce TCR clustering, they were able to probe the effect of signal duration on TCR signaling at time scales relevant for the on and off rates of an individual TCR (**Figure 2**, left). Calcium imaging revealed that T cells failed to respond to fast clustering dynamics (short cluster half-life). When clusters persisted beyond a threshold duration, the T cell response was inversely proportional to the cluster half-life. Using mathematical modeling, they estimated the response threshold to correspond to a half-life of approximately 8 seconds. Optogenetic approaches provide a controlled platform for testing predictions of KPR models by isolating the contribution of binding duration to signaling output. These experiments align with a substantial body of work using altered peptide ligands and TCR mutants, which demonstrate that the T cell response depends strongly on the dwell-time of the TCR-pMHC interaction (58, 61). Mathematical and computational studies further show how sequential signaling steps can amplify small differences in binding duration to produce robust antigen discrimination (75–77). Recent single-molecule and FRET-based measurements confirm that the dwell-time of TCR-pMHC interactions is a critical determinant of signaling outcome, beyond simple receptor occupancy or equilibrium affinity (62, 78). Collectively, these experimental and theoretical approaches demonstrate that T cells can decode the dynamics of receptor-ligand interactions to discriminate between antigens.

Downstream of the TCR, the activation of T cells requires the integration of many small signals over time (e.g., single molecule phosphorylations, step increases in cytoplasmic calcium (79, 80), proteolytic cleavage, nuclear translocation). Since each signaling intermediate (e.g., phosphorylated protein) exhibits different kinetics and has a limited lifetime before returning to its resting state, the system as a whole will be sensitive to the dynamics of the antigen-dependent stimulus. Experimentally, optogenetic approaches have also been used to explore the impact of TCR stimulation dynamics at the chemical reaction timescales of several minutes. O’Donoghue et al. (81) used a CAR-T cell optogenetic model to demonstrate that T cell signaling was sensitive to the frequency of T cell stimulation, independently of the total magnitude of stimulus. They found that pulses with a timescale of about 25 minutes have a reduced activation effect compared to shorter or longer pulses. The mechanism of the frequency detector in the T cell was not fully determined, but was shown to arise from the intracellular protein interaction network (**Figure 2**, middle), specifically the signaling pathway downstream of ERK activation. Jaeger et al. (82) also demonstrated frequency-dependent effects of the stimulus. In particular, they show that T helper cells and cytotoxic T cells respond differentially to pulsatile signals. They further demonstrate that cytokine production also varies between continuous and pulsatile stimulations.

**Figure 4:**
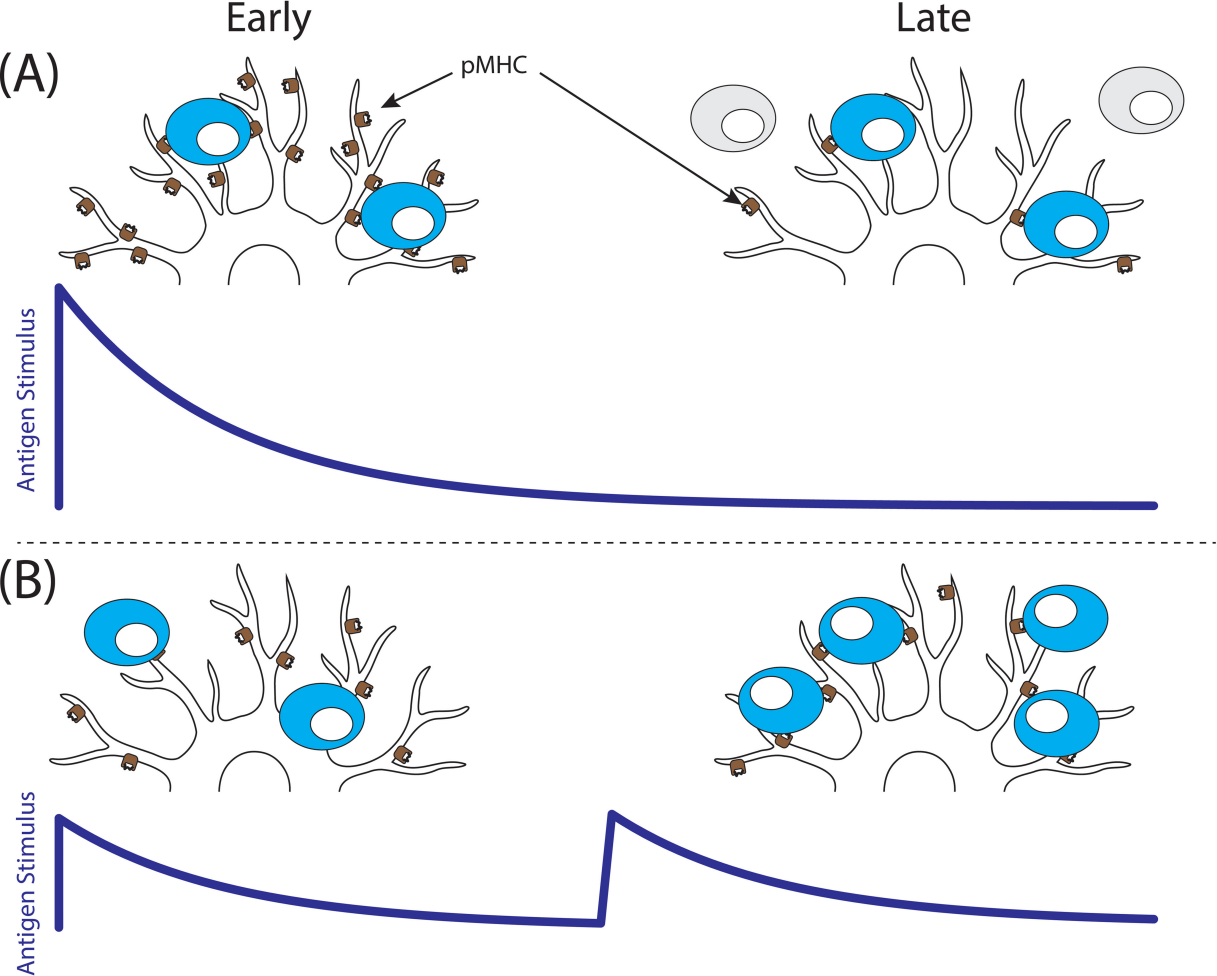
Competition for pMHC on the surface of antigen presenting cells can result in different T cell responses to different temporal patterns of antigen stimulation. Early after a single exposure to antigen **(A)**, pMHC levels are high, and the response is limited by T cell numbers. However, late in the response, pMHC levels fall, and T cells compete for limited access. If a second dose of antigen is introduced **(B)**, levels of pMHC rise again, and more T cells can be stimulated.

All the mechanisms above operate at a cell-autonomous level. The response to antigen dynamics has also been investigated at the level of cell circuits (**Figure 2**, right), focusing on interactions between different populations of immune cells. Mathematical models for the dynamics of antigen removal, either through T cell consumption (43) or T cell independent decay (44), have analyzed the effect of these dynamics on T cell proliferation. These studies propose a model in which T cell proliferation is driven directly by exposure to antigen. As antigen levels drop, the late response is limited by inter-T cell competition (**Figure 4A**). These models predict the experimentally observed power-law dependence of T cell expansion on initial T cell number. Mayer et al. (44) further show that the degradation dynamics of antigens, driven by pMHC or antigen-presenting cell turnover, yield a power-law dependence of T cell expansion on affinity, thereby enhancing the specificity of the response. This model further suggests that providing antigen stimulation at lower levels over extended periods will enhance the T cell expansion. Maintaining antigen availability at later times reduces inter-T cell competition and thus optimizes their proliferative capacity (**Figure 4B**). Furthermore, the model specifically predicts that an exponential increase in antigen levels would optimize T cell responses, consistent with data on mouse immunization (83).

**Figure 5:**
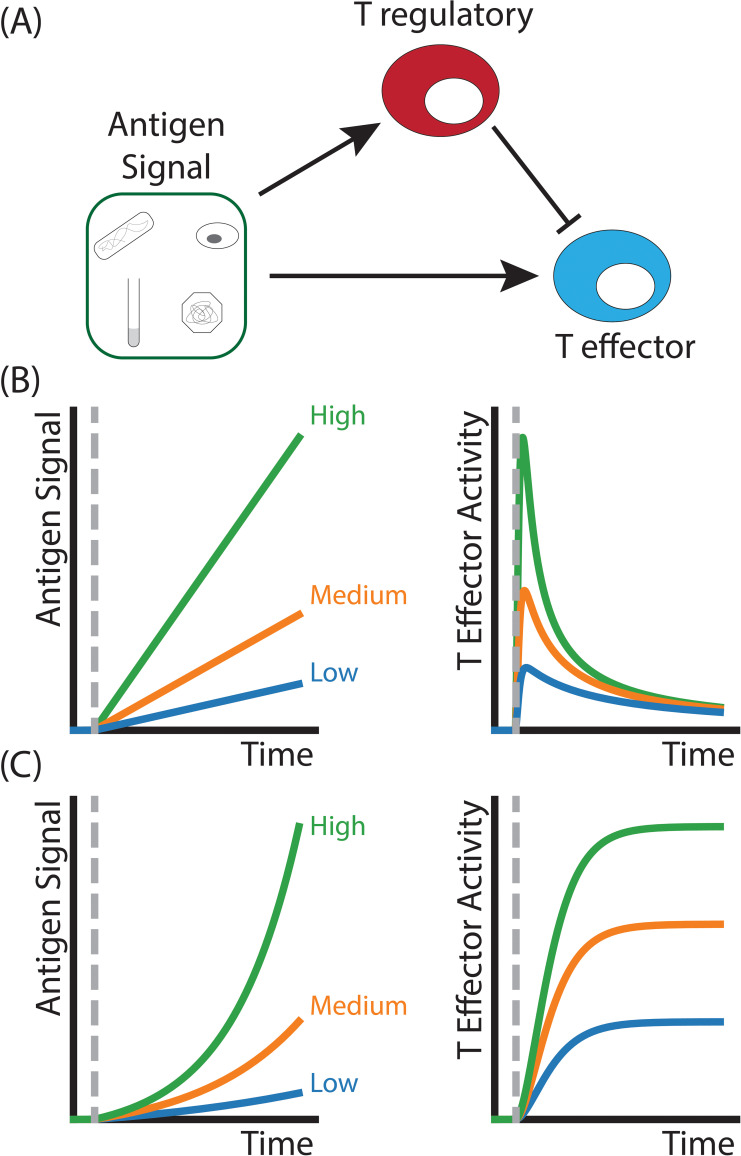
Incoherent feedforward loops can sense and respond to different dynamical patterns in T cell stimulation. Antigen can drive both an effector and a regulatory T cell response, leading to an incoherent feedforward network pattern **(A)**. When antigen changes linearly (**B**, left), the network responds transiently and returns to the same baseline (**B**, right; dynamical compensation). In response to exponentially increasing antigen concentrations (**C** left), the model predicts a sustained response whose magnitude varies depending on the exponential growth rate (**C** right). Line colors represent different total antigen levels, as indicated.

Mechanisms incorporating regulatory networks of T cells have also been shown to be sensitive to antigen dynamics (16). This model (**Figure 5A**) is based on the idea that an antigen can drive activation of both effector cells and regulatory T cells (84). This results in the well-studied network motif, often referred to as an incoherent feedforward loop (85). Such a loop comprises a fast-activating sequence and a parallel slower inhibitory sequence. When this motif is stimulated by a pulse, a short response is followed by adaptation of the system and return to baseline. As shown by Sontag, this architecture is inherently sensitive to the dynamics of the antigen. For pulses or linearly increasing stimuli, the system responds only transiently, returning to the same basal level (**Figure 5B**). However, for exponentially increasing antigen signals the model predicts a sustained long-term response (**Figure 5C**). It is interesting to note that effector cells acquire many of the properties of T regulatory cells subsequent to activation, including impaired ability to secrete cytokines, and expression of high levels of the IL2R alpha chain (86), CTLA-4 (87) and PD1 (88, 89). Thus, a more general incoherent feedforward topology could incorporate regulation by effector T cells at later times post-activation, acting to regulate and switch off the immune response. By incorporating a negative feedback of the effector T cells on the stimuli itself, the model can result in either activation or tolerance depending on the parameters and the antigen exponential rate. This model has been used to explain self/non-self discrimination, as well as the partial and usually ineffective natural immune response to cancer.

While antigen dynamics significantly influence the magnitude and duration of T cell responses, effector differentiation is the result of a complex, multi-factorial program. Beyond antigenic cues, which are particularly critical during early activation phases, T cell fate decisions are shaped by programmed transcriptional circuits, cytokine environments, co-stimulatory pathways, and metabolic constraints (90–94). These signals integrate with antigen-dependent inputs to regulate T cell expansion and differentiation (11). Consequently, while models based on antigen dynamics provide a powerful framework for understanding rate-sensing, they represent one layer of a broader integrative regulatory network that governs T cell differentiation and long-term function.

## Conclusions

In this review, we consider the dynamics of the antigen landscape and the ability of T cells to sense and respond to temporal signals. We have identified different timescales, from fractions of seconds to multiple days, over which the antigenic signal can vary. These timescales arise from different biological processes, which include the dynamics of antigen, the dynamics of pMHC, and the dynamics of TCR-pMHC interaction (**Figure 1**). We have also brought together evidence that suggests that T cells respond not just to the amount of signal but also explicitly to its dynamics, with different mechanisms being evoked for different timescales (**Figures 2**, **3**, **4**). The binding and unbinding of TCR and pMHC complexes leads to a thresholded response where pulses shorter than a few seconds do not elicit an activation response (**Figure 3**). Above the threshold, downstream protein networks interpret the frequency of the signal to determine the amplitude and type of the response by activating specific T cell subtypes. On long timescales of multiple days, the exact shape of the antigenic stimulus can result in suboptimal activation, where ideal stimulation arises for exponentially increasing signals (**Figures 4**, **5**). Population-level networks allow T cells to read out the exponential rate of antigen growth and result in tolerance or elimination of the challenge in a rate-dependent manner.

Studying T cell responses under dynamic antigen stimulation is key to advancing our understanding of the immune system. While the immune system is widely appreciated as highly dynamic, the specific role of antigen dynamics in T cell activation has received relatively little attention and remains supported by limited experimental and theoretical work. In particular, intermediate timescales, ranging from minutes to hours, remain poorly characterized. Increased attention to antigen dynamics may also help address the challenges of model selection. For example, De Boer and Perelson (43) and Mayer et al. (44) develop distinct models that both fit the same data set, but incorporate quite distinct assumptions regarding the regulation of antigen dynamics.

Optimizing the dynamics of antigen delivery offers a powerful approach for therapeutic intervention. In autoimmunity and allergy, controlled low-dose or sustained antigen exposure may promote tolerance by favoring regulatory over effector responses. Conversely, in vaccinology, replacing traditional bolus administration with temporally controlled antigen exposure, for example, through increasing amounts of antigen delivery over several days, can enhance immune responses (83, 95). Such temporal patterns more closely match the kinetics of natural infection, where antigen levels increase over similar timescales (**Figure 1**). These observations suggest that immune responses can be tuned not only by antigen dose but also by its temporal profile, highlighting antigen dynamics as a key design parameter in immunotherapy. Bridging these empirical observations with a mechanistic understanding of how T cells decode temporal antigen signals remains an important challenge. Consequently, developing quantitative and predictive dynamical models will have significant implications for immuno-therapeutic strategies, requiring additional basic research.
